# Balloon bursting: transurethral puncture of a Foley catheter balloon

**DOI:** 10.1093/jscr/rjaa588

**Published:** 2021-01-29

**Authors:** Stephanie F Smith, Anup Sengupta

**Affiliations:** Department of Urology, Norfolk and Norwich University Hospital, Norwich, UK; Department of Urology, West Suffolk Hospital, Bury St Edmunds, UK

## Abstract

We describe the case of a 76-year-old male who presented with a retained Foley catheter. On the initial attempt to deflate the Foley catheter with a syringe attached to the inflation port, no fluid was aspirated. Using a flexible cystoscope passed alongside the catheter, the balloon was punctured under direct vision with an intravesical needle passed through the cystoscope working channel. We discuss this innovative and safe method for balloon puncture via the transurethral route that can be achieved in the urology outpatient or endoscopy suite.

## INTRODUCTION

The Foley catheter, originally described in 1929, is the most common type of indwelling urinary catheter. It consists of a flexible tube containing a lumen that drains urine, and another lumen connected to an inflatable balloon, typically holding 10 ml, that secures the catheter in position. To remove the catheter, the balloon is deflated by aspiration of the side port, allowing its removal. Catheter removal is most often straightforward; however, on occasion it can be challenging and result in a retained catheter. Isolated case reports and case series of retained Foley catheters have been described in the literature, but accurate estimates of its incidence are unknown.

The causes of a retained catheter include a balloon that does not deflate and/or significant external encrustation deposits on the catheter causing resistance. Patients with encrusted retained catheters may describe bladder spasms, urine bypassing the catheter and suprapubic pain. Encrustations consist of crystalline deposits, largely comprising struvite and/or apatite [1]. Infection and catheter colonization by urease-producing bacteria is the predominant cause of crystalline biofilms. The urease-producing bacteria most commonly associated with encrustations are *Proteus mirabilis* [2], but other examples include *Klebsiella pneunoniae*, *Pseudomonas aeruginosa* and *Morganella morganii* [3]. It is thought that up to 50% of patients having long-term catheters will experience catheter encrustation [4].

In this case report, we describe the case of a 76-year-old male who presented with a retained Foley catheter due to inability to deflate the balloon.

## CASE REPORT

A 76-year-old gentleman presented to the urology outpatient clinic for a routine change of his long-term 14F urinary catheter, indicated for incomplete voiding secondary to benign prostatic hyperplasia. Due to his medical co-morbidities, namely severe chronic obstructive pulmonary disease, ischemic cardiac disease and hypertension, a long-term catheter had been chosen rather than pursuing surgical intervention. On examination, he had a prostatic size of ~100 cc.

On the initial attempt to deflate the Foley catheter with a syringe attached to the inflation port, no fluid was aspirated. Over-inflation of the balloon was attempted up to 30 ml of sterile water, but this was unsuccessful and caused the patient significant discomfort. Guidewire balloon puncture using a polytetrafluoroethylene (PTFE) guidewire inserted into the catheter outflow port was then attempted, which was also unsuccessful.

Ultrasound-guided needle puncture was attempted initially via the transrectal route, which was unsuccessful. Needle puncture was also attempted using the suprapubic route under ultrasound guidance in the radiology department, also unsuccessfully.

Finally, a flexible cystoscope was passed transurethrally alongside the urinary catheter. An intravesical needle was inserted via the working channel and this was used to successfully puncture the catheter balloon under direct vision. The needle used was an Olympus™ single-use flexicystoscope needle (MAJ-656), which had a tip length of 4 mm and working length of 105 cm.

The procedure was performed with gentamicin antibiotic prophylaxis. The retrieved balloon was significantly encrusted. The patient was clinically well post-procedure and discharged after a short period of observation. The patient was given advice to increase his oral fluid intake, and his next urethral catheter change was planned for 2-month time in the urology department.

## DISCUSSION

It is not an uncommon scenario for the duty urologist to be asked to review a patient who has a retained catheter due to its balloon not deflating. A systematic approach to the causes of non-deflation of the catheter balloon was described by Shaprio *et al*. [5]. The problem may arise due to malfunction of the inflation valve (preventing return of fluid filling the balloon), physical damage to the inflation channel or obstruction [5] and encrustation [6].

Balloon rupture may be achieved by passing a wire through the inflation port, chemical methods or physical puncture (transurethral, percutaneous or endoscopic) [5]. If there is severe encrustation that precludes the above methods, holmium laser stone fragmentation [7] and extracorporeal shock wave lithotripsy [8] are troubleshooting methods that have also been described.

Hollingsworth *et al.* [9] published a case series of 13 patients with retained Foley catheter due to inability to deflate the catheter balloon. They described success in 15% of patients in deflating the balloon by passing a wire through the balloon port. They were successful in deflating the balloon in 31% of patients by cutting the catheter (with or without subsequent aspiration), and in 31% of patients, they required invasive maneuvers for extra-luminal balloon puncture via a transvaginal, transurethral or suprapubic approach.

The benefit of the technique we have employed, transurethral puncture, is it can be performed under local anesthesia in the outpatient or endoscopy suite setting. Hamilton *et al.* [10] describe a case report of using flexible video cystoscopy-guided balloon puncture. Their case is remarkably similar to our patient, in which a 70-year-old man with a long-term indwelling catheter attended for his routine 3-monthly catheter change and routine methods of deflation were unsuccessful. They passed a biopsy forceps through the flexible cystoscope working channel to deflate the balloon [10].

The recommended time between long-term catheter replacement varies depending on local policies. National Institute for Health and Care Excellence (NICE) guidelines (CG139 2012) recommend that catheter changes should be guided by the manufacturer’s instruction or when clinically necessary, but the frequency may be modified in anticipation of obstruction based on previous change requirements. The specific catheter used in this case report (Rusch™ Brillant AquaFlate silicone) has a manufacturer recommendation that the catheter may be used up to 12 weeks. It was decided to bring forward the next routine catheter change appointment to 8 weeks, as a shorter time interval would reduce the likelihood of significant encrustation from crystalline biofilms.

In conclusion, using a cystoscopic-guided intravesical needle is a safe and effective method of achieving balloon puncture for a retained Foley catheter that can be achieved in the urology outpatient or endoscopy clinic setting. Timely catheter exchanges and maintaining good fluid intake may reduce the risk of catheter encrustation.
